# *Phomopsis longanae* Chi-Induced Change in ROS Metabolism and Its Relation to Pericarp Browning and Disease Development of Harvested Longan Fruit

**DOI:** 10.3389/fmicb.2018.02466

**Published:** 2018-10-16

**Authors:** Hui Wang, Yihui Chen, Hetong Lin, Junzheng Sun, Yifen Lin, Mengshi Lin

**Affiliations:** ^1^Institute of Postharvest Technology of Agricultural Products, College of Food Science, Fujian Agriculture and Forestry University, Fuzhou, China; ^2^Food Science Program, Division of Food Systems and Bioengineering, University of Missouri, Columbia, MO, United States

**Keywords:** longan fruit, disease development, pericarp browning, reactive oxygen species (ROS), ROS metabolism, ROS scavenging ability, *Phomopsis longanae* Chi

## Abstract

*Phomopsis longanae* Chi is a major pathogenic fungus that infects harvested longan fruit. This study aimed to investigate the effects of *P. longanae* on reactive oxygen species (ROS) metabolism and its relation to the pericarp browning and disease development of harvested longan fruit during storage at 28°C and 90% relative humidity. Results showed that compared to the control longans, *P. longanae*-inoculated longans displayed higher indexes of pericarp browning and fruit disease, higher O_2_^-.^ generation rate, higher accumulation of malondialdehyde (MDA), lower contents of glutathione (GSH) and ascorbic acid (AsA), lower 1,1-diphenyl-2-picrylhydrazyl (DPPH) radical scavenging ability and reducing power in pericarp. In addition, *P. longanae*-infected longans exhibited higher activities of superoxide dismutase (SOD), catalase (CAT), and ascorbate peroxidase (APX) in the first 2 days of storage, and lower activities of SOD, CAT, and APX during storage day 2–5 than those in the control longans. These findings indicated that pericarp browning and disease development of *P. longanae*-infected longan fruit might be the result of the reducing ROS scavenging ability and the increasing O_2_^-.^ generation rate, which might lead to the peroxidation of membrane lipid, the loss of compartmentalization in longan pericarp cells, and subsequently cause polyphenol oxidase (PPO) and peroxidase (POD) to contact with phenolic substrates which result in enzymatic browning of longan pericarp, as well as cause the decrease of disease resistance to *P. longanae* and stimulate disease development of harvested longan fruit.

## Introduction

Longan (*Dimocarpus longan* Lour.) is a popular tropical fruit famous for its appealing flavor and abundant nutritional ingredients (Lin et al., 2001; Chen et al., 2015). However, harvested longan fruits deteriorate rapidly due to water loss, injury, energy deficiency, pathogen infection, or damage caused by reactive oxygen, resulting in pericarp browning, decline of fruit quality and rot (Holcroft et al., 2005). Pericarp browning is a vital factor affecting edible quality and commercial value of postharvest longan fruits seriously (Jiang et al., 2002). There are abundant phenolic compounds in longan pericarp tissue (Prasad et al., 2009; Yang et al., 2011). Researches indicated that pericarp browning of postharvest longan fruit was mainly due to the formation of browning substances resulting from enzymatic browning (Wang et al., 2015; Lin et al., 2017b).

Reactive oxygen species (ROS), such as superoxide anion (O_2_^-.^)and hydrogen peroxide (H_2_O_2_), was reported to cause pericarp browning of fruits (Shah et al., 2017). Overaccumulation of ROS could lead to peroxidation of membrane lipid, loss of compartmentalization of cells and organelles, and formation of browning substances when peroxidase (POD) and polyphenol oxidase (PPO) contact with phenols (Lin Y.F. et al., 2016, Lin et al., 2017a; Sun et al., 2018). There are active oxygen-scavenging enzymes including catalase (CAT), superoxide dismutase (SOD), and ascorbate peroxidase (APX) (Jiang et al., 2015; Luo et al., 2015; Xu et al., 2017; Chen et al., 2019). Moreover, plant cells contain non-enzymatic endogenous antioxidant substances, including glutathione (GSH) and ascorbic acid (AsA) (Sun et al., 2018).

Fungal infection is a main problem in quality keeping for harvested longan fruits (Chen et al., 2014; Zhang et al., 2017, 2018). *Phomopsis longanae* Chi is a major fungus infecting postharvest longans (Chen et al., 2014, 2018b). *P. longanae*-inoculation could induce disease development and pericarp browning of postharvest longans (Chen et al., 2018a). However, there is no comprehensive understanding about the mechanisms of disease development and pericarp browning of postharvest longans induced by *P. longanae*.

This study aimed to analyze effects of *P. longanae*-inoculation on production rate of O_2_^-.^, content of malondialdehyde (MDA) (resulting from oxidative damage of membrane lipids), activities of CAT, SOD, and APX, levels of GSH and AsA, reducing power and 1,1-diphenyl-2-picrylhydrazyl (DPPH) radical scavenging ability, and to explore mechanisms of pericarp browning and disease development of postharvest longans induced by *P. longanae*-infection.

## Materials and Methods

### Materials and Treatments

The spore suspension of *P. longanae* with the concentration of ×10^4^ spores mL^-1^ was prepared according to Chen et al. (2014).

Longan (*Dimocarpus longan* Lour. cv. Fuyan) fruits at commercial maturity were harvested from an orchard in Quanzhou, Fujian, China, thereafter carried back by a refrigerated truck to our laboratory in less than 3 h.

Fruits were selected with absence of injury or diseases, and uniform size, color and maturity. and absence of injury, blemishes, insect pests or diseases. Fruits were surface-sterilized by dipped in 0.5% NaClO for 10 s, thereafter were air dried. Then 30,150 longans were used for experiment. Among them, 150 fruits were selected for used for analysis on the harvest day (day 0). The rest 3, 000 longans were divided into a control group (1,500 longans) and a *P. longanae*-inoculated group (1,500 longans). Longans in control group were dipped in sterile deionized water for 5 min, while longans in another group used for inoculation were treated by *P. longanae* spore suspension instead of sterile deionized water for 5 min. After air-dried, all the treated fruits were packed (50 fruits/bag) in polyethylene bags (0.015-mm-thick). Each treatment contains 30 bags. Then the fruits were stored under the same storage condition of 28°C and 90% relative humidity. 150 fruits (3 bags) from each treatment were randomly selected at each storage day to determine the physiological and biochemical indexes and their relations to longan pericarp browning and disease development caused by *P. longanae* (Chen et al., 2018a).

### O_2_^-.^ Generation Rate and MDA Content

One gram of pericarp tissue from 10 longans was used for analysis of O_2_^-.^ the generation rate according to previously reported method (Lin et al., 2014). The O_2_^-.^ generating rate was represented as nmol g^-1^ min^-1^.

One gram of pericarp tissue from 10 longans was used for measurement of MDA content according to previously reported method (Lin et al., 2014). The MDA content was represented as mmol g^-1^.

### Activities of SOD, CAT, and APX

The activities of SOD, CAT, and APX, and the protein content were determined using one gram of pericarp from 10 longans, respectively, referring to previously reported method (Sun et al., 2018). Activities of these enzymes were represented as U mg^-1^ protein.

### Contents of GSH and AsA

One gram of pericarp tissue from 10 longans was used to measure contents of GSH and AsA, respectively, according to previously reported method (Sun et al., 2018). The contents of GSH and AsA were represented as mg kg^-1^.

### DPPH Radical Scavenging Activity and Reducing Power

One gram of pericarp tissue from 10 longans was used to measure DPPH radical scavenging activity and reducing power, respectively, with previously reported method (Sun et al., 2018). The DPPH radical scavenging ability and reducing power were represented as % and g kg^-1^, respectively.

### Statistical Analysis

All experiments were repeated for three times. Data were represented in form of means ± standard error. Statistical analyses were performed for analyzing the data by SPSS Statistics (version 17.0). Statistical differences were assessed with a significant level when *P*-value less than 0.05.

## Results

### Effects of *P. longanae* Infection on O_2_^-.^ Generation Rate and MDA Content in Pericarp of Harvested Longan Fruit

**FIGURE 1:**
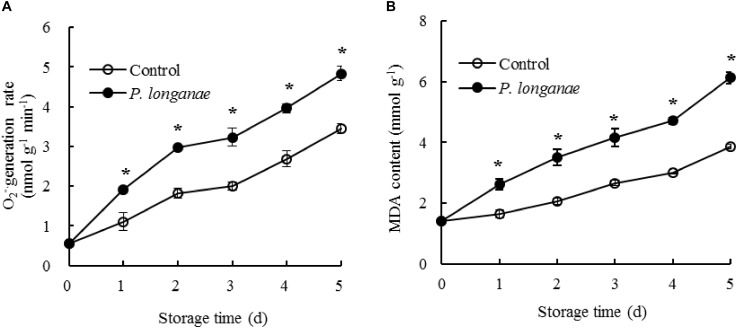
Effects on O_2_^-.^ generation rate **(A)** and MDA content **(B)** in pericarp of postharvest longan fruits inoculated by *P. longanae*. The symbol “^∗^” indicates difference significant between control and *P. longanae*-inoculated fruit (*P* < 0.05).

The generation rate of O_2_^-.^ in pericarp tissue of the control longan fruits rose rapidly within 0–2 days, then a slow increase from 2 to 3 days, thereafter a quick increase during 3–5 days of storage (**Figure 1A**). The O_2_^-.^ generation rate in *P. longanae*-inoculated longan fruits showed a trend similar as the control longans. Moreover, the O_2_^-.^ generation rate was higher (*P* < 0.05) in postharvest longans inoculated by *P. longanae* compared to the control longans during 1–5 days of storage.

The content of MDA in pericarp tissue of the control longan fruits presented a gradual rise in storage days 0–5 (**Figure 1B**). While the content of MDA in postharvest longans inoculated by *P. longanae* showed a rapid rise from 0 day of storage. For example, the MDA content of *P. longanae*-inoculated longan fruits increased about 4-fold from storage day 0 to day 5. Statistical comparison suggested that the MDA content in postharvest longans inoculated by *P. longanae* was higher (*P* < 0.05) compared to the control longan fruits during 1–5 days of storage.

### Effects of *P. longanae* Infection on Activities of SOD, CAT, and APX in Pericarp of Harvested Longan Fruit

**FIGURE 2:**
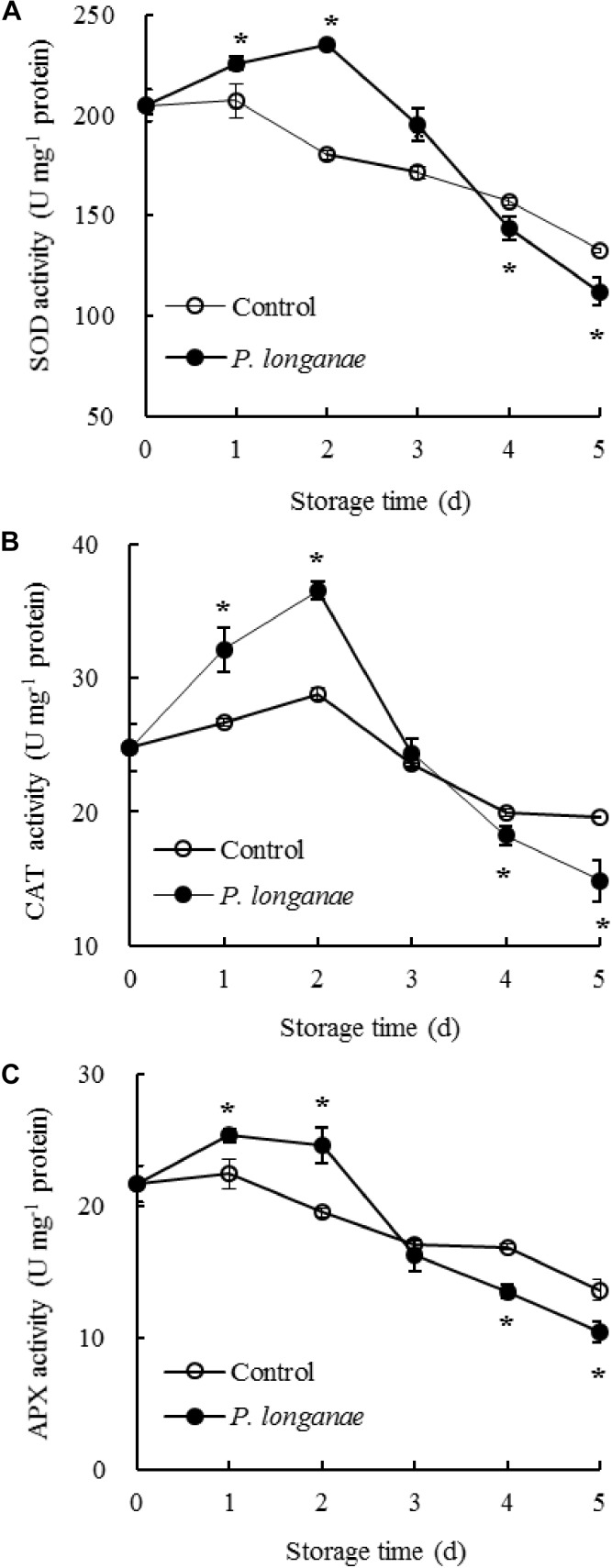
Effects on activities of SOD **(A)**, CAT **(B)**, and APX **(C)** in pericarp of postharvest longans inoculated by *P. longanae*. The symbol “^∗^” indicates difference significant between control and *P. longanae*-inoculated fruit (*P* < 0.05).

The SOD activity changed little during 0–1 day of storage, and thereafter declined rapidly in pericarp tissue of the control longan fruits (**Figure 2A**). While SOD activity increased within 0–2 days of storage, thereafter decreased quickly in longans inoculated by *P. longanae*. Compared to control longans, the *P. longanae*-inoculation treated longans showed a higher (*P* < 0.05) and a lower (*P* < 0.05) SOD activity during 1–3 and 4–5 days, respectively.

The CAT activity in the control longan pericarp tissue rose slightly during the first 0–2 days, then declined rapidly after 2 days of storage (**Figure 2B**). Whereas, the CAT activity showed a dramatical rise in the first 0–2 days of storage and declined sharply thereafter in *P. longanae*-inoculated longans. Further comparison showed CAT activity was higher (*P* < 0.05) in pericarp tissue of postharvest longans inoculated by *P. longanae* within 0–2 days of storage, and lower (*P* < 0.05) on the storage day 5 compared to the control longans.

**Figure 2C** showed that in control longan pericarp tissue the APX activity changed little within 0–1 day, followed by a reduction from 1 to 5 days. However, the APX activity in *P. longanae*-inoculated longan fruits showed a noticeably rise during 0–1 day, and changed little during 1–2 days, then declined rapidly during 2–5 days. Furthermore, in pericarp tissue of longans inoculated by *P. longanae*, APX activity was higher (*P* < 0.05) within the first 2 days, and lower (*P* < 0.05) during 4–5 days of storage compared to control longans.

### Effects of *P. longanae* Infection on Contents of GSH and AsA in Pericarp of Harvested Longan Fruit

**FIGURE 3:**
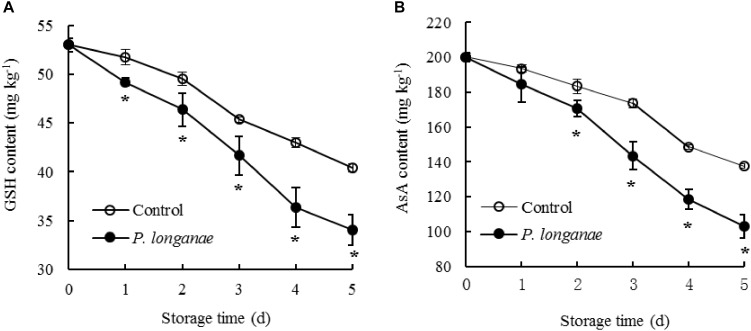
Effects on contents of GSH **(A)** and AsA **(B)** in pericarp of postharvest longans inoculated by *P. longanae*. The symbol “^∗^” indicates difference significant between control and *P. longanae*-inoculated fruit (*P* < 0.05).

Contents of GSH and AsA reduced rapidly as storage time prolonging in pericarp tissue of the control longan fruit (**Figure 3**). Compared to control longan fruit, *P. longanae*-inoculated longans displayed faster decline of contents of GSH and AsA from 0 to 5 days. Moreover, the contents of GSH and ASA were lower (*P* < 0.05) in longans inoculated by *P. longanae* compared to the control longans during 2–5 days of storage.

### Effects of *P. longanae* Infection on DPPH Radical Scavenging Ability and Reducing Power in Pericarp of Harvested Longan Fruit

**FIGURE 4:**
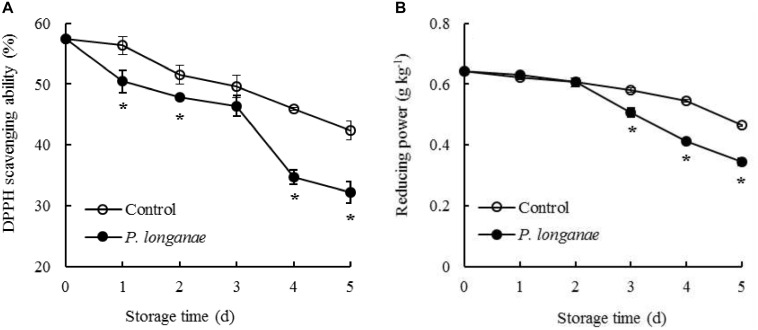
Effects on DPPH radical scavenging ability **(A)** and reducing power **(B)** of postharvest longans inoculated by *P. longanae*. The symbol “^∗^” indicates difference significant between control and *P. longanae*-inoculated fruit (*P* < 0.05).

The DPPH radical scavenging ability in control longan pericarp tissue exhibited a slightly decrease from 0 to 1 day, followed by a faster decreased from 1 to 5 days of storage (**Figure 4A**). Whereas, the DPPH radical scavenging ability in longans inoculated by *P. longanae* exhibited a rapid decline from 0 d, thereafter a decrease during 1–3 days, a sharp reduction during 3–4 days, then a fast decline during 4–5 days. Furthermore, there was a lower (*P* < 0.05) DPPH radical scavenging ability in longans inoculated by *P. longanae* compared to the control longans within 1–5 days except storage day 3.

The reducing power decreased slowly within 0–4 days in pericarp tissue of the control longan fruits, thereafter declined rapidly from 4 to 5 days of storage (**Figure 4B**). While the reducing power in longans inoculated by *P. longanae* decreased slightly during 0–2 days, then declined remarkably from 4.06 g kg^-1^ on storage day 3 to 2.76 g kg^-1^ on day 5. Compared to the control longans, the *P. longanae*-inoculation treated longan fruits showed a lower (*P* < 0.05) reducing power in pericarp tissue during 3–5 days of storage.

## Discussion

Longan fruit is prone to turn to pericarp browning and diseased after harvest, which results in a decline in fruit quality and commercial value, and is the main limitation of its long-time storage and long-distant transport (Sun et al., 2018). Pathogen infection is a major reason accounting for disease and pericarp browning of postharvest longans (Chen et al., 2014). Disease development and pericarp browning of postharvest longans was thought to be related to reactive oxygen metabolism disorder and accumulation of ROS (Chomkitichai et al., 2014; Lin Y.F. et al., 2016). It has been reported that H_2_O_2_ and O_2_^-.^ accumulated during the process of disease and pericarp browning development in harvested longans (Lin et al., 2017a). Chen et al. (2018a) reported that compared to the control longans, *P. longanae*-inoculated longans displayed higher indexes of pericarp browning and fruit disease. In present work, results showed that *P. longanae*-infection induced the rise of O_2_^-.^ generation rate (**Figure 1A**). During 0–2 days of storage, the rise of O_2_^-.^ generation rate might be related with the defensive reaction against the infection of *P. longanae* (Sun et al., 2018). MDA is a product resulting from oxidative damage of membrane lipids (Wang et al., 2013, 2014, 2015). In this study, the content of MDA accumulated as storage time prolonging (**Figure 1B**). However, *P. longanae*-infection enhanced the accumulation of MDA, indicating that the biomembrane system was seriously damaged in *P. longanae*-inoculate longan fruit.

In plant cells, there are ROS scavenging enzymes playing crucial roles in protecting cells from oxidative stress (Bloknina et al., 2003; Luo et al., 2015; Xu et al., 2017; Chen et al., 2019). SOD can catalyze two O_2_^-.^ to H_2_O_2_ and O_2_ (Duan et al., 2007). Both CAT and APX could catalyze H_2_O_2_ to H_2_O and O_2_ (Yi et al., 2010). Under normal circumstances, the ROS is in a dynamic equilibrium between generation and scavenging. However, when the equilibrium is broken under stress conditions, ROS would be accumulated (Yi et al., 2008; Sun et al., 2018). The increase of ROS account for membrane lipid peroxidation and cellular compartmentalization lost, leading to the disease increase and enzymatic browning resulting from oxidation of phenolic substrates by PPO and POD (Sun et al., 2010; Lin et al., 2013, 2017c, 2018; Jiang et al., 2018). It has been reported that the enhanced activities of CAT, SOD, and APX might be caused by pathogen infection in early infection stage to scavenge excessed ROS in harvested longans (Lin et al., 2015). Whereas, the activities of CAT, SOD, and APX decreased with prolonged storage time, leading to the increase of O_2_^-.^ content, peroxidation of membrane lipid and increased browning of harvested longans (Sun et al., 2018).

In present study, activities of SOD, CAT, and APX showed rise within 0–2 days in *P. longanae*-inoculated longans (**Figure 2**). Meanwhile, the generation rate of O_2_^-.^ increase quickly (**Figure 1A**). Thus, the enhanced activities of SOD, CAT, and APX when O_2_^-.^ generation rate rise in early stage (0–2 days of storage) of *P. longanae*-infection might be a defense to reduce contents of ROS. Besides, in longans infected by *P. longanae* activities of CAT, SOD, and APX decreased rapidly during 2–5 days of storage while the O_2_^-.^ generation rate still exhibited a trend of increase. This indicated that the equilibrium between ROS scavenging enzymes and ROS was broken during 2–5 days of storage, resulting in the rise of O_2_^-.^ generation rate, membrane lipid peroxidation, and accumulation of MDA.

In addition, non-enzymatic antioxidant substances, including GSH and AsA, exist in fruit tissues, playing important roles in ROS elimination (Jiang et al., 2015, 2018). AsA and GSH participate in the reaction of catalyzing H_2_O_2_ to H_2_O by APX (Lin et al., 2017a). Our results presented that contents of GSH and AsA decreased quickly in longans inoculated by *P. longanae* and showed a lower (*P* < 0.05) compared with the control longan fruits during 2–5 days of storage (**Figure 3**). It can be inferred that *P. longanae*-infection induced decrease of GSH and AsA contents in pericarp tissue of postharvest longan fruits, which might account for the increase of O_2_^-.^ generation rate during storage.

Besides, there is another free radical-scavenging system for inhibiting lipid peroxidation (Sun et al., 2018). The reducing power and DPPH radical scavenging activity were generally tested to analysis the antioxidant activity (Sun et al., 2007; Lin et al., 2017a). The browning in pathogen infected fruit pericarp tissue was thought to be correlated with the reduced reducing power and the decline of DPPH radical scavenging activity (Sun et al., 2018). In this research, the *P. longanae*-inoculated longans showed lower reducing power and DPPH radical scavenging ability compared to control longans in late storage period (4–5 days). Meanwhile, the O_2_^-.^ generation rate (**Figure 1A**), pericarp browning developed faster in *P. longanae*-inoculated longan compared with control longans (Chen et al., 2018a). This suggested that the rising O_2_^-.^ generation rate and browning index of *P. longanae*-inoculated longans might due to the reduced antioxidant activity.

## Conclusion

**FIGURE 5:**
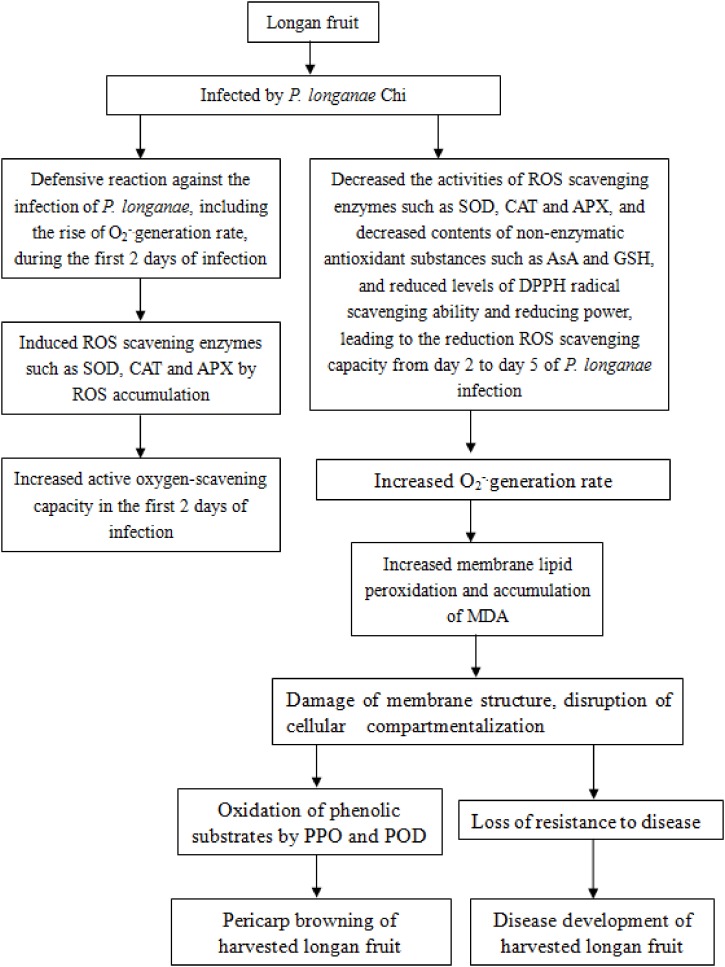
The probable mechanism of *P. longanae*-induced pericarp browning and disease development of postharvest longan fruit via acting on ROS metabolism.

In conclusion, the disruption of reactive oxygen scavenging system by *P. longanae* inoculation might be a vital reason causing pericarp browning accelerated and disease increased of postharvest longan fruits. The increasing O_2_^-.^ generation rate and content of MDA in longans infected by *P. longanae* might be the result of the decreased ROS scavenging enzyme activities, the reduced the contents of non-enzymatic antioxidant substances, the declined reducing power, and the lowered DPPH radical scavenging ability, which leads to the peroxidation of membrane lipid, the loss of compartmentalization in longan pericarp cells, and subsequently cause PPO and POD to contact with phenolic substrates, in turn, result in enzymatic browning of longan pericarp, as well as cause the decrease of disease resistance to *P. longanae* and stimulate disease development of harvested longan fruit. The probable mechanism of *P. longanae*-induced pericarp browning and disease development of postharvest longan fruit via acting on ROS metabolism was demonstrated in **Figure 5**.

## Author Contributions

HW and YC performed the experiments. HL designed the research. JS and YL conducted the experiments and analyzed the data. HW and YC wrote the manuscript. HL revised the manuscript. ML edited English language of the manuscript. All authors have approved the submission and publication of the manuscript.

## Conflict of Interest Statement

The authors declare that the research was conducted in the absence of any commercial or financial relationships that could be construed as a potential conflict of interest.
